# Interannual hydrological changes affect plant communities across different elevation zones in plateau lakeshores: insights from Lake Erhai

**DOI:** 10.3389/fpls.2024.1439772

**Published:** 2024-11-14

**Authors:** Feng Zhu, Jing Yuan, Zeying Hou, Xia Guo, Wanxue Liao, Shenglin Yang, Zhaosheng Chu

**Affiliations:** ^1^ College of Water Sciences, Beijing Normal University, Beijing, China; ^2^ National Engineering Laboratory for Lake Pollution Control and Ecological Restoration, Chinese Research Academy of Environmental Sciences, Beijing, China; ^3^ Construction Project Environmental Impact Assessment and Audit Center of Dali Bai Autonomous Prefecture, Dali, Yunnan, China

**Keywords:** plateau lakeshore, wet-dry alternation, soil nutrients, plant diversity, structural equation modeling

## Abstract

The relationship between wetland water level changes and plant community has been a research hotspot. However, the gradient changes and critical influencing factors of plateau lakeshore plants and soils during wet-dry alternation remain unclear. Here, we studied the variations in plants and soils along the Erhai lakeshore across three elevation ranges (1965.0-1965.3m, 1965.3-1965.6m, and 1965.6-1966.4m) during flooding and drought years. Our research aimed to elucidate the interrelationships and mechanisms among hydrology, soil properties, and plant dynamics. The results showed that (1) In drought years, the Shannon-Wiener index of plants significantly decreased across the three elevation ranges, and other plant diversity indices, biomass, and coverage also decreased to varying degrees; (2) except for soil pH, soil water (SW) and nutrient content decreased to varying degrees in the drought year; (3) SW was the primary factor influencing plant biomass, coverage, and diversity in the 1965.0-1965.3m and 1965.3-1965.6m ranges; nitrate nitrogen, C/N ratio, total phosphorus were the primary factors in the 1965.6-1966.4m ranges. The results of structural equation modeling revealed a significant and strong correlation between SW and plant biomass, coverage, and soil pH. This suggests that changes in SW directly impacted plant biomass accumulation, subsequently affecting coverage, and also played a role in regulating soil pH. This study identified the effects of hydrological inter-annual changes on plant communities and highlighted SW as a crucial driver. The strategies proposed in the results protect and improve the diversity and stability of lake ecosystems in Lake Erhai and other similar lakes.

## 1 Introduction

The lakeshore zone, serving as a transition zone between lake and terrestrial ecosystems, are a hotspot for biodiversity research, but a sensitive area susceptible to periodic water level changes (Wang et al., 2022; Zhang et al., 2015). The lakeshore zone is periodically exposed to water due to water-level fluctuations, which the typical hydrological characteristics, so any tiny changes in the hydrological condition heavily affect the vegetation composition and structures. Under the dual influence of climate change and human activities, water level fluctuation varied rapidly and difficultly predicted, posing a severe challenge to the maintenance of biodiversity in lakeshores (Fluet-Chouinard et al., 2023; Xiong et al., 2023). Climate change-induced floods and droughts alter the hydrological conditions of lakes, thereby affecting the growth ranges of wetland plants (Wan et al., 2019). In particular, the alternation of dry and wet soil changes in lakeshores caused by hydrological differences can result in a series of cascading effects on wetland plants, such as species colonization and expansion (Crisman et al., 2014), root depth and architecture (Fan et al., 2017; Zhang et al., 2018), and seed germination rate (Nishihiro et al., 2004; Wang et al., 2023a). Therefore, it has become more urgent to explore the effects of hydrological conditions on the plant communities in the lakeshore zone to address the impacts of climate change and human activities.

The dynamic nature of hydrology frequently serves as a primary driver in the succession of wetland plant communities, potentially directly influencing the species composition and structure of the plant community (Deng et al., 2014; Sun et al., 2022). The highest and lowest water levels in wetlands are critical factors that determine the habitat range of plant communities (Chapin and Paige, 2013). For example, different dominant plant species occupy distinct water level ranges, emergent plants (such as N*elumbo nucifera, Typha orientalis, Phragmites australis*) probably are more adapted to higher water levels (Qin et al., 2021), while *Carex* rapidly germinates and grows in low-water levels (Yuan et al., 2017). Moreover, the diverse habitats formed under varying water level conditions significantly contribute to the diversity of wetland plants, with water level fluctuations emerging as a key influencing factor (Fu et al., 2022; Riis and Hawes, 2002; Thiet, 2002). Influenced by periodic changes in hydrology, the duration and extent of flooding affect the growth rate and nutrient absorption efficiency of wetland plants, consequently influencing the accumulation of plant biomass (Lawniczak et al., 2010; Luo et al., 2016). Favorable hydrological conditions result in higher biomass accumulation by wetland plants, enhancing the fixation and storage of carbon in soils (Liu et al., 2015). In summary, although previous studies have demonstrated that lakeshore plants respond differently to water level fluctuations, they have primarily focused on single water level conditions or specific plant communities. There remains a lack of research on how extreme hydrological events affect plant communities across different elevation ranges in the lakeshore zone.

The hydrological processes in the lakeshore zone not only shape the soil environment, but directly influence the transport and enrichment of soil nutrients (Feng et al., 2020). Firstly, water level fluctuations affect soil water content and regulate soil pH (Yu et al., 2023). Secondly, the ecotone hydrological processes alter soil nutrient content by regulating nutrient retention time, organic matter accumulation and microbial community structure (Ren et al., 2022; Sollie and Verhoeven, 2008). For example, long-term flooded lakeshore, due to nutrient-rich water and gentle flow, dissolved nutrients are retained after sufficient biochemical reactions, facilitating nutrient accumulation in the flooded zone (Bernal et al., 2013; Rücker and Schrautzer, 2010). Conversely, lakeshore at higher elevations are less susceptible to water level fluctuations, resulting in relatively limited soil water content (Zhang et al., 2022). Wetland soils provide essential nutrient support for plant growth, including total nitrogen, total phosphorus, organic matter, and other components, thus significantly influencing plant community composition and diversity (Fan et al., 2019; Ma et al., 2021; Song et al., 2023). However, the gradient changes and interrelationships between plants and soils in the lakeshore zones of plateau lakes during wet-dry alternation remain unclear.

Lake Erhai is one of the nine plateau lakes in the Yunnan Province of China, with rich biological resources, and is an important ecological protection area and biodiversity conservation area in China (Chen and Wu, 2020; Li et al., 2018b). In recent years, the Erhai lakeshore zone has experienced severe ecosystem damage due to the combined effects of lake eutrophication and human activities (tourism and land use), leading to a drastic decline in vegetation coverage (Wang et al., 2023b; Wang et al., 2015). To restore the damaged lakeshore wetlands of Lake Erhai, optimize the structure of plant communities and recover plant coverage, a series of protection, management and ecological construction projects have been implemented in Lake Erhai since 2017 (Lin et al., 2020). However, a comprehensive investigation of the plant communities in the lakeshore zone is lacking. The water level in Lake Erhai has been artificially regulated since the construction of hydroelectric power plants in the 1980s. The water level is low in the summer (May to June) and high in the autumn (September to November), where the seasonal variation of water level is opposite to the hydrological rhythm of most natural lakes. In particular, as the operating water level of Lake Erhai decreased to the legal minimum of 1964.30 m in 2023 (DBAPPG, 2023), it is crucial to explore how this decrease impacts the structure and diversity of the lakeshore wetland plant communities.

Therefore, we conducted plant investigations and soil sampling in the Erhai lakeshore wetlands during both the flooding year (2022) and the drought year (2023). The main purposes of this study were to (1) reveal the changes in soil properties, plant community composition and diversity at different elevations during two hydrological years; (2) explain the interrelationships between “hydrological changes - soil properties - plant responses” under different flooding scenarios. The research results can provide a theoretical basis for managing water level suitability at the Erhai lakeshore zone and proposing strategies to protect and improve the diversity and stability of lake ecosystems.

## 2 Materials and methods

### 2.1 Study site

**Figure 1:**
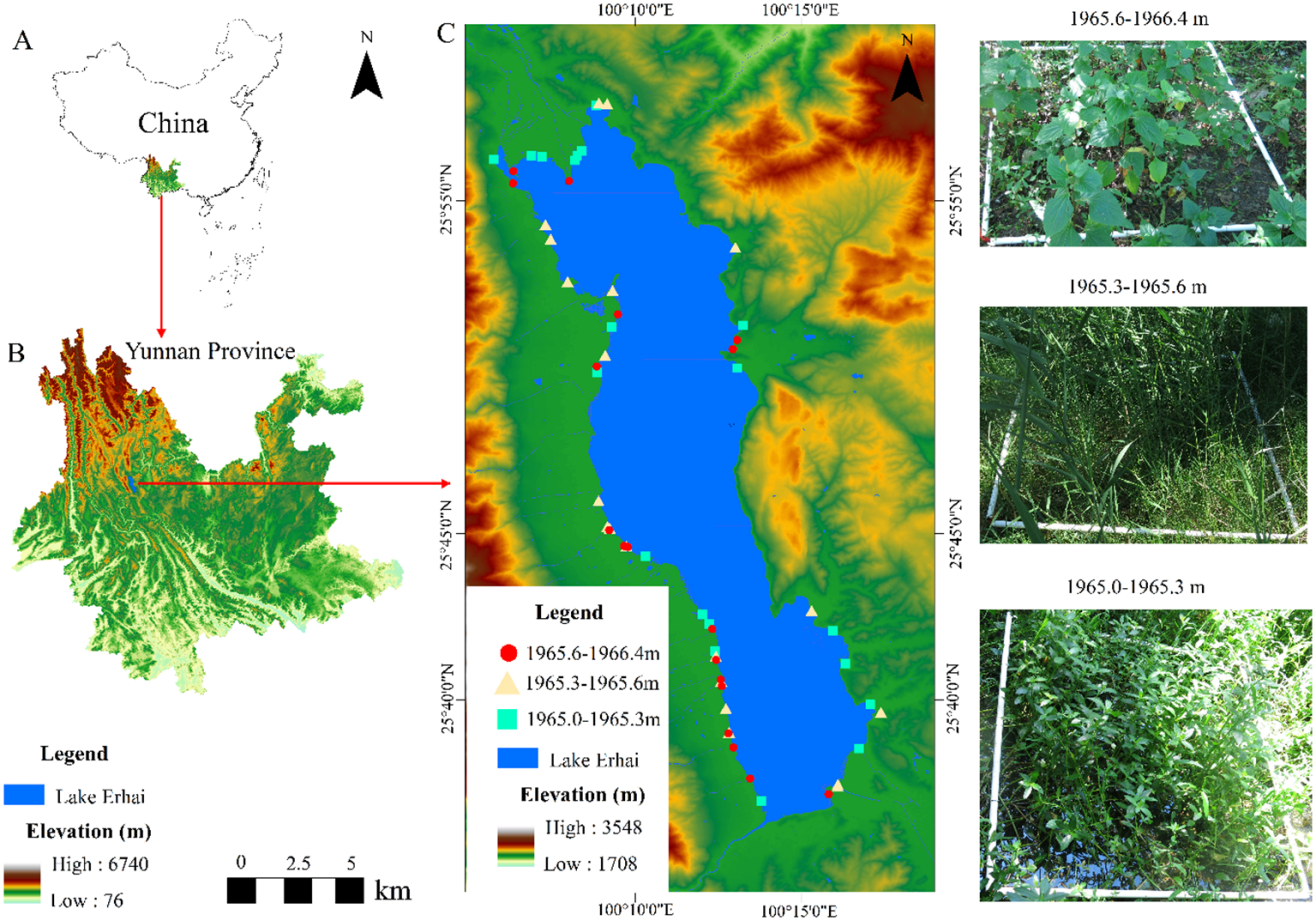
Location of the Lake Erhai based on a 30m resolution Digital Elevation Model **(A , B)**. Coordinates of sample plots along the Lakeshore of Lake Erhai **(C)**.

This study was conducted at Lake Erhai (25°36′~25°58′ N, 100°06~100°18′ E), the second largest plateau freshwater lake located in Yunnan Province, China (**Figure 1**). It is a typical subtropical Plateau Lake with a total area of 252 km², the lowest operating water level is 1964.30 m and the highest is 1966.00 m (i.e., the Yellow Sea’s elevation) (Gong et al., 2023). Over the past two decades, interannual and seasonal water level variations have been 1.0 and 1.2 m, respectively (Wen et al., 2021; Yuan et al., 2024). The water level plays a crucial role in the biological community and ecological service function of Lake Erhai, and the regulation of water level in the lake area is primarily based on precipitation and the inflow of water from tributaries into the lake (Fu et al., 2013; Wen et al., 2021; Yang et al., 2021). In Lake Erhai basin, the slope overflows are relatively high, the tributaries are numerous but short, and the rivers and agricultural ditches were the main channel of terrestrial pollutants, mainly agricultural and rural non-point source pollution into the lake (Cao et al., 2024; Peng et al., 2024). Water quality in Lake Erhai has been improved significantly with improved local government management, and now the lake is in a mesotrophic situation. The region experiences a subtropical monsoon climate, characterized by an average annual temperature of 15°C. Precipitation amounts to 870 mm during the rainy season (May to October) and 170 mm during the dry season (November to April).

### 2.2 Field investigation and sampling

Field investigations and soil sampling were conducted in July 2022 and 2023. We selected 58 fixed tree samples (20 × 20 m) along the entire shoreline of Lake Erhai. Latitude and longitude coordinates were recorded using a hand-held GPS locator, and red rope markers were spray-painted or hung at the four corners of each sample for reference. Additionally, a 1m × 1m herbaceous plant quadrat was set up at the corners and the center of each tree sample, and the name, number, coverage, height, and aboveground biomass of each species were recorded. This study focused on plants, therefore only measured data within plant quadrats were used.

After clearing the aboveground biomass in each quadrat, five soil samples (0-20 cm) were collected by the S-shape sampling method, mixed into one composite sample for physicochemical properties analysis (Liu et al., 2018). The aboveground biomass within each quadrat was weighed after being dried for 48 h at 65°C. The water surface relative elevation of each sample plot was measured by a level instrument, and combined with the water level on the day of the investigation, the elevation of the sample plot was calculated (Shen et al., 2019).

### 2.3 Laboratory analyses

All soil samples were transported back to the laboratory. Some fresh soil was used to determine soil water content (SW), while another portion was stored at 4°C for the determination of ammonia nitrogen (NH_4_^+^-N) and nitrate nitrogen (NO_3_^−^-N). The remaining samples were cold-dried, ground, passed through a 0.149 mm nylon sieve, and then stored for further analyses, including total phosphorus (TP), total nitrogen (TN), C/N ratio, soil organic matter (SOM), and pH.

Determination of soil water content by thermostat drying method (Zhang et al., 2005). NH_4_^+^-N and NO_3_^−^-N were extracted with a KCl solution and then determined by spectrophotometric methods (UV-1900i). TP according to the molybdenum blue colorimetric method was measured with a UV/visible spectrophotometer (UV-1900i) (Lan et al., 2019). Samples were pre-treated and total nitrogen (TN%) and C/N ratio were measured by an Elemental Analyzer (Vario Macro Cube, Germany). Soil organic matter (SOM) determined by potassium dichromate volumetric method (Ji, 2005). Soil pH was determined using a pH meter in a 1:2.5 water: soil mixture.

### 2.4 Data analyses

We used average monthly water level to determine water level differences for the 2022 and 2023, and regression analyses to test the relationship between sample plot elevations and flooding days (FD). The importance value represents the significance of a species within its community and is utilized to assess its dominance (Hertling and Lubke, 1999). In our study, species with an importance value of ≥ 0.01 were defined as dominant species and calculated as follows (Lili et al., 2011):


(1) $$\begin{array}{c} \text{IV}=\;(\text{relative height }+\text{ relative coverage }\\ +\text{ relative frequency }+\text{ relative biomass})/4 \end{array}$$

Shannon-Wiener index (*H*), Simpson Index (*D*), Pielou Evenness index (*E*) and Patrick index (*R*) were used as indicators of species diversity and were calculated as follows (Strong, 2016):


(2) $$H\;=\;-\sum_{i=1}^{s}P_{i}\text{ln}P_{i}$$

(3) $$D=1\;-\sum_{i=1}^{s}P_{i}^{2}$$

(4) $$\text{E}\;=\;\frac{\text{H}}{\text{ln(S)}}$$

(5) R=S

where *S* is the total number of species (species richness) recorded in each sample plot and *P_i_* corresponds to the relative abundance of each species.

The 58 sample plots were evenly divided into three ranges (*N* = number of sample plots) based on elevation from lowest to highest: 1965.0-1965.3m (*N*=20), 1965.3-1965.6m (*N*=20), and 1965.6-1966.4m (*N*=18). We used independent samples t-tests and Mann-Whitney U-tests to assess inter-annual differences in diversity indices, species richness, biomass, coverage and soil physicochemical properties across the three elevation ranges. A redundancy analysis (RDA) was used to explain the multivariate relationships between plant community characteristics (biomass, coverage, and diversity indices) and environmental factors (flooding days and soil physicochemical properties) at three elevation ranges for two sampling years.

Structural equation modeling (SEM) was used to explore the relationships between soil (water content, pH, and nutrients) and plants (biomass, coverage, and diversity). The first step in SEM requires developing a conceptual model of factor structure hypothesis based on *a priori* and theoretical foundations. Path coefficients were calculated using maximum likelihood estimation, and the model was optimized by removing observed variables from the prior model based on modification indices (Delgado-Baquerizo et al., 2013; Wei et al., 2013; Zhao et al., 2017). Key fit indices were used to confirm the completion of the model fit.

RDA analysis was completed using CANOCO Version 5.0 (Plant Research International, Wageningen, The Netherlands). SEM model construction and analysis were completed in AMOS 26.0 (IBM SPSS, Inc.). The other statistical analyses were completed using SPSS 27.0 (SPSS Inc., Chicago, USA).

## 3 Results

### 3.1 Hydrological conditions

**Figure 2:**
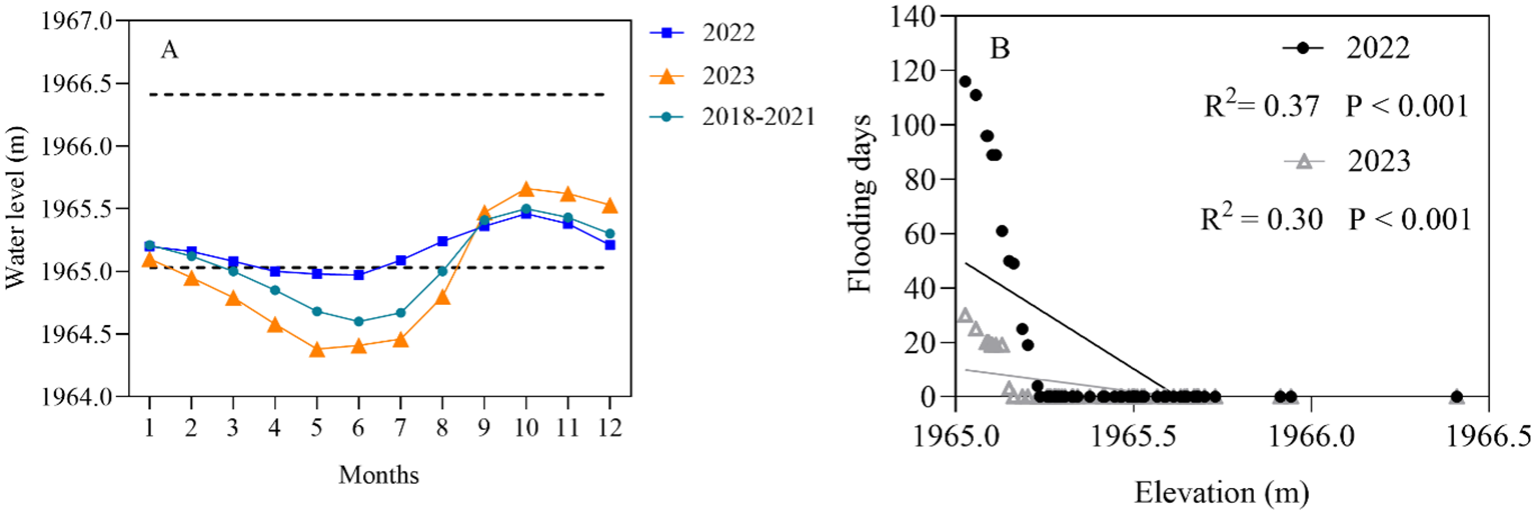
**(A)** The average monthly water level of Lake Erhai in 2022, 2023, and 2018-2021. The dashed lines indicate the upper and lower ranges of sample plot elevations. The values are mean ± SE. **(B)** Correlation analysis between the elevation of each sample plot and flood days from January to July in 2022 and 2023.

The seasonal variation in the water level of Lake Erhai exhibited a unimodal pattern, with lower in summer and higher in winter. Compared to the January-July average water level (AWL) of 1964.87m from 2018-2021, the AWL was 0.2m higher in 2022, while it was 0.21m lower in 2023 (**Figure 2A**). In this study, the total number of flooding days was calculated for each sample plot during January-July 2022 and 2023, respectively. The results indicated that the range of flooding days was 4-116 days in 2022, whereas it declined to 3-30 days in 2023. Therefore, there was a significant positive correlation between the elevation of the sample plots and the flooding days. Within the elevation range of 1965.0-1965.3m, the flooding days were significantly higher in 2022 compared to 2023; however, the difference gradually decreased with increasing elevation (**Figure 2B**).

### 3.2 Species composition and diversity

A total of 104 species belonging to 38 families and 85 genera were surveyed for vascular plants in 2022. The plant families with the largest number of species were Asteraceae (*N*=19, 18.3%), Poaceae (*N*=19, 18.3%), Polygonaceae (*N*=6, 5.8%), Balsaminaceae (*N*=4, 3.8%), and Lamiaceae (*N*=3, 2.9%). The number of species declined significantly in 2023, with a total of 89 species from 34 families and 70 genera surveyed. Compared to 2022, the number of Asteraceae species decreased to 18, Poaceae increased to 20 species, Balsaminaceae decreased to 1 species, while Polygonaceae and Lamiaceae remained unchanged.

**Figure 3:**
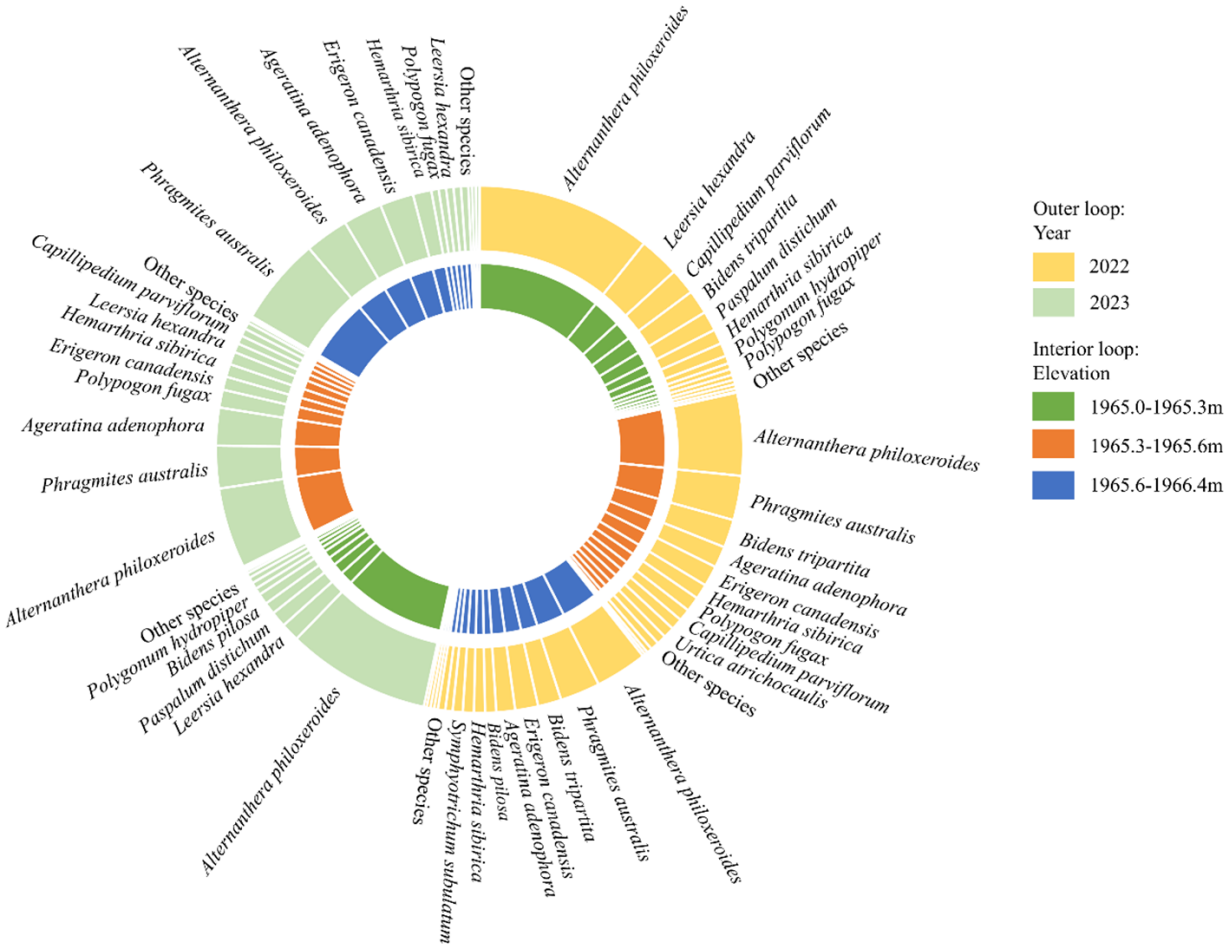
Interannual variation of importance value index for the dominant species between elevations. The colors of the interior loop represent three elevation ranges: 1965.0-1965.3m, 1965.3-1965.6m, and 1965.6-1966.4m, while the colors of the outer loop indicate the investigated years: 2022 and 2023. The areas of the loop segments are proportional to the importance value of each species.

At the dominant species level, there was inter-annual variability in the distribution and importance value of herbaceous dominant species at different elevations (**Figure 3**). The main dominant herbaceous species is *Alternanthera philoxeroides* (IV2022: 0.47 ± 0.17; IV2023: 0.39 ± 0.22), which is most densely distributed in the range of 1965.0-1965.3m. The importance value of hygrophilous plants such as *Paspalum distichum、Hemarthria sibirica、Leersia hexandra、Capillipedium parviflorum、Bidens tripartita、Polygonum hydropiper* in 2023 was reduced in the range of 1965.0-1965.3m, with these species being less distributed in the range of 1965.6-1966.4m. In total, the dominant species in the range 1965.0-1965.3m were 13 in 2022 and 9 in 2023; those in the range 1965.3-1965.6m were 13 in 2022 and 11 in 2023; and those in the range 1965.6-1966.4m were 14 in 2022 and 13 in 2023. The inter-annual variation of all dominant species importance values is listed in **Appendix A1**.

**Figure 4:**
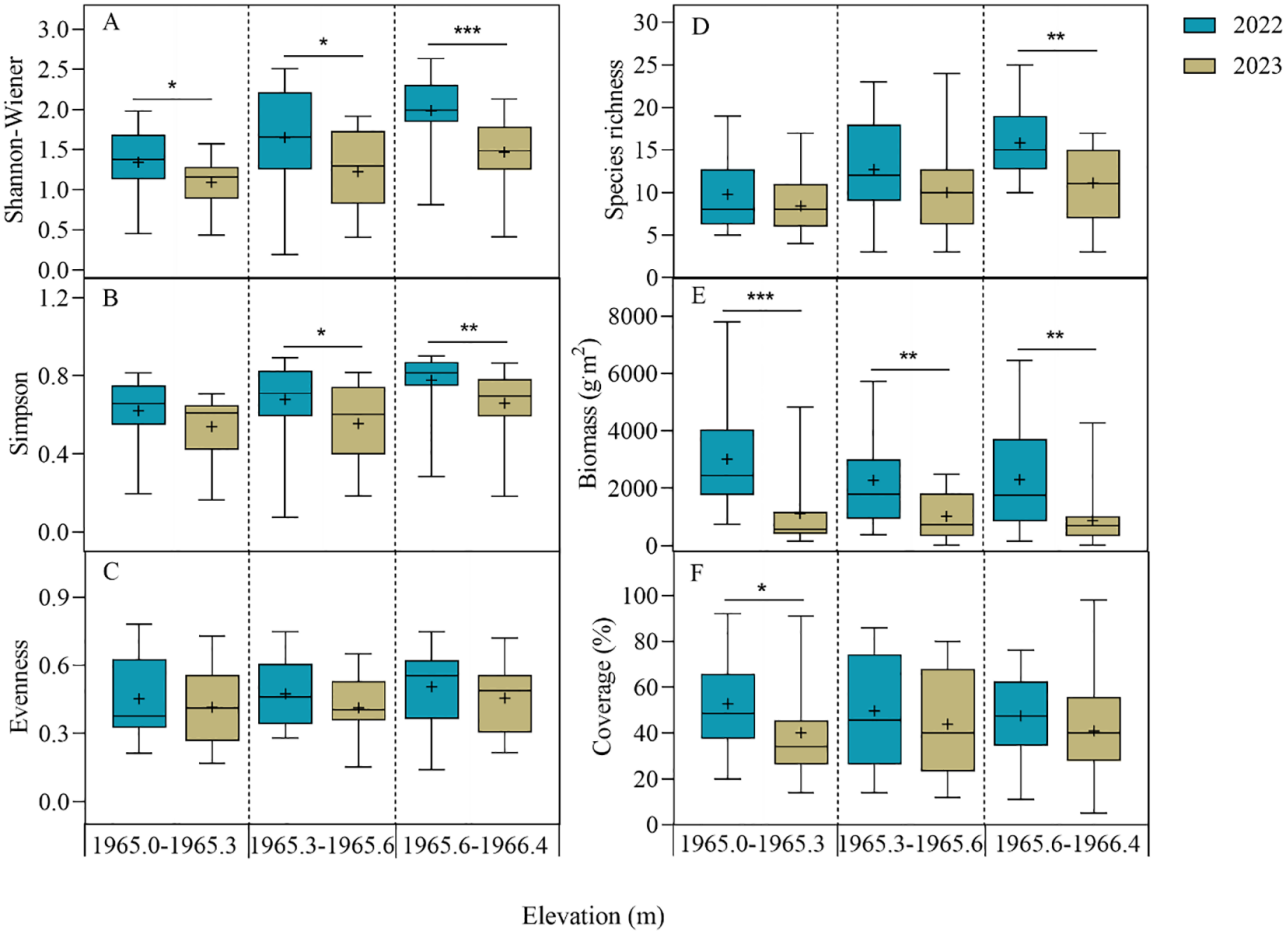
Interannual variation in diversity indices **(A–D)**, biomass **(E)** and coverage **(F)** at different elevation ranges in 2022 and 2023. The values are mean ± SE, + indicates the mean value. Significant differences are indicated by symbols: * *P* < 0.05, ** *P* < 0.01, *** *P* < 0.001; no symbol, non-significant difference.

At the diversity index level, each of the four indices showed varying degrees of decrease across different elevation ranges in 2023 (**Figures 4A–D**). The Shannon-Wiener index was highest at the 1965.6-1966.4 m, with a significant decrease of 25.76%, and lowest at the 1965.0-1965.3 m, with a significant decrease of 18.66%. Similarly, the Simpson index was highest at the 1965.6-1966.4 m, with a significant decrease of 14.29%, and lowest at the 1965.0-1965.3 m, with a decrease of 12.90%. Evenness exhibited no significant decrease across the three elevation ranges. Species richness changed consistent with the Shannon-Wiener, Simpson, and Evenness indices, such that the elevation ranges with higher species richness also had higher diversity indices. Biomass and coverage were both highest at 1965.0-1965.3 m, biomass decreased by 62.85% and coverage decreased by 23.80% in 2023. In summary, plant diversity was highest, while biomass and coverage were relatively low at 1965.6-1966.4 m.

### 3.3 Soil physical - chemical properties

**Figure 5:**
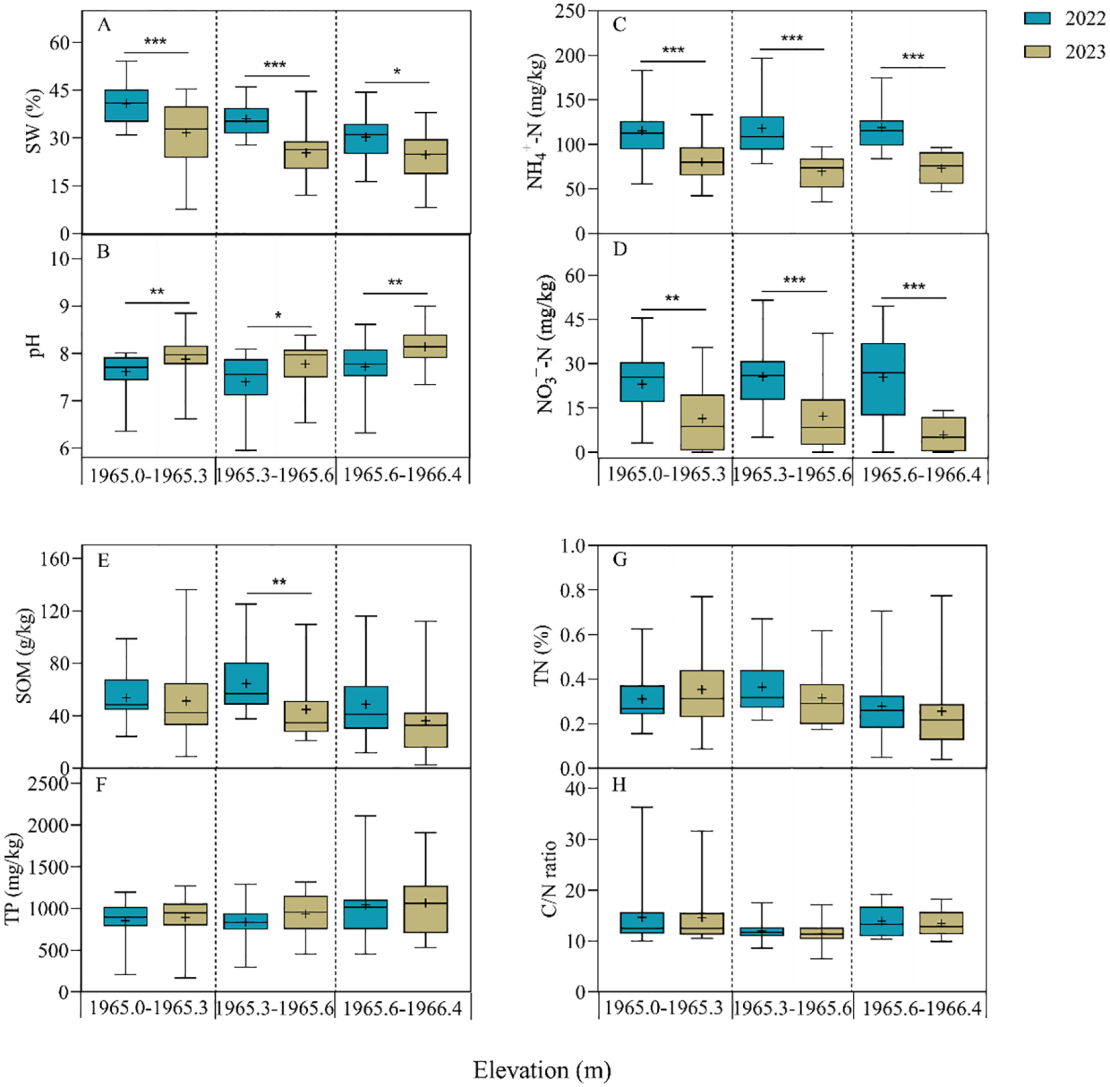
Variations in soil physicochemical properties at different elevation ranges in 2022 and 2023. SW, Soil water content **(A)**; pH **(B)**; NH_4_^+^-N: ammonia nitrogen **(C)**; NO_3_^−^-N: nitrate nitrogen **(D)**; SOM: soil organic matter **(E)**; TP: total phosphorus **(F)**; TN: total nitrogen **(G)**; C/N ratio **(H)**. The values are mean ± SE, + indicates the mean value. Significant differences are indicated by symbols: * *P*< 0.05, ** *P*< 0.01, *** *P*< 0.001; no symbol, non-significant difference.

Soil physicochemical properties were significantly changed during the two hydrologic years, and their variations at different elevation ranges differed in 2023 (**Figure 5**). The SW was highest at the 1965.0-1965.3 m, with a significant decrease of 22.30%, and lowest at the 1965.6-1966.4 m, with a significant decrease of 18.20%. Conversely, the pH was highest at the 1965.6-1966.4 m, with a significant increase of 5.44%, and lowest at the 1965.0-1965.3 m, with an increase of 3.41%. NH_4_^+^-N and NO_3_^−^-N significantly decreased across all three elevation ranges. NH_4_^+^-N decreased the most at 1965.3-1965.6 m, by 40.81%; NO_3_^−^-N decreased the most at 1965.6-1966.4 m, by 76.96%. SOM significantly decreased by 30.45% at 1965.3-1965.6 m, while TP, TN, and the C/N ratio showed no significant differences across the three elevation ranges. In summary, the results indicate that drought significantly influenced SW, pH, NH_4_^+^-N, and NO_3_^−^-N, while showing no significant effects on other soil nutrients.

### 3.4 Relationships between hydrology, plants and soil

**Figure 6:**
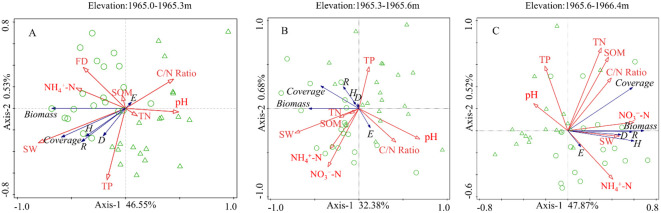
Redundancy analysis for different elevation ranges. **(A)** 1965.0-1965.3m. **(B)** 1965.3-1965.6m. **(C)** 1965.6-1966.4m. SW, soil water content; FD, flooding days; NH_4_^+^-N, ammonia nitrogen; NO_3_^−^-N, nitrate nitrogen; SOM, soil organic matter; TP, total phosphorus; TN, total nitrogen. *D*: Simpson index; *H*: Shannon-Wiener index; *E*: Pielou Evenness index. *R*: species richness. Green circle: 2022; green triangle: 2023.

The redundancy analysis ranking results revealed significant correlations between environmental factors and plant diversity indices, coverage, biomass, and species richness across the three elevation ranges. Moreover, the degree of explanation varied among the different environmental factors (**Figure 6**; **Table 1**). At 1965.0-1965.3m, the first and second principal component axis explained 46.55% and 0.53%, respectively. Among them, SW, TN, SOM and C/N ratio explained 30.3%, 7.2%, 6.4% and 1.5%, respectively (**Figure 6A**). At 1965.3-1965.6m, the first and second principal component axis explained 32.38% and 0.68%, respectively. Among them, SW, pH and SOM explained 17.0%, 7.3% and 2.4%, respectively (**Figure 6B**). At 1965.6-1966.4m, the first and second principal component axis explained 47.87% and 0.52%, respectively. Among them, NO_3_^−^-N, C/N ratio, TP and SW explained 17.1%, 8.0%, 7.1% and 6.9%, respectively (**Figure 6C**). In summary, SW was the crucial influencing factor at the 1965.0-1965.3 m and 1965.3-1965.6 m elevation ranges, whereas soil nutrients were crucial at the 1965.6-1966.4 m.

**Table 1:** RDA analysis results of explanatory variables for different elevation ranges.

Explanatory variables	% variance explained	*P* value	*F* ratio
1965.0-1965.3m			
SW	30.3	0.002	16.5
TN	7.2	0.032	4.3
SOM	6.4	0.04	4.1
C/N ratio	1.5	0.55	1
NH_4_^+^-N	0.4	0.632	0.2
pH	0.3	0.702	0.2
TP	0.3	0.79	0.2
FD	0.1	0.802	<0.1
1965.3-1965.6m			
SW	17	0.008	7.8
pH	7.3	0.062	3.6
SOM	2.4	0.294	1.2
TP	2.1	0.346	1
C/N ratio	1.9	0.298	1
TN	1.2	0.426	0.6
NO_3_^−^-N	0.3	0.71	0.1
NH_4_^+^-N	<0.1	0.884	<0.1
1965.6-1966.4m			
NO_3_^−^-N	17.1	0.008	7
C/N ratio	8	0.064	4.1
TP	7.1	0.064	3.3
SW	6.9	0.078	3
TN	3.2	0.208	1.7
SOM	2.5	0.214	1.3
pH	3	0.224	1.6
NH_4_^+^-N	<0.1	0.852	<0.1

SW, Soil water content; TN, total nitrogen; SOM, soil organic matter; TP, total phosphorus; FD, flooding days; NH_4_^+^-N, ammonia nitrogen; NO_3_^−^-N, nitrate nitrogen.

**Figure 7:**
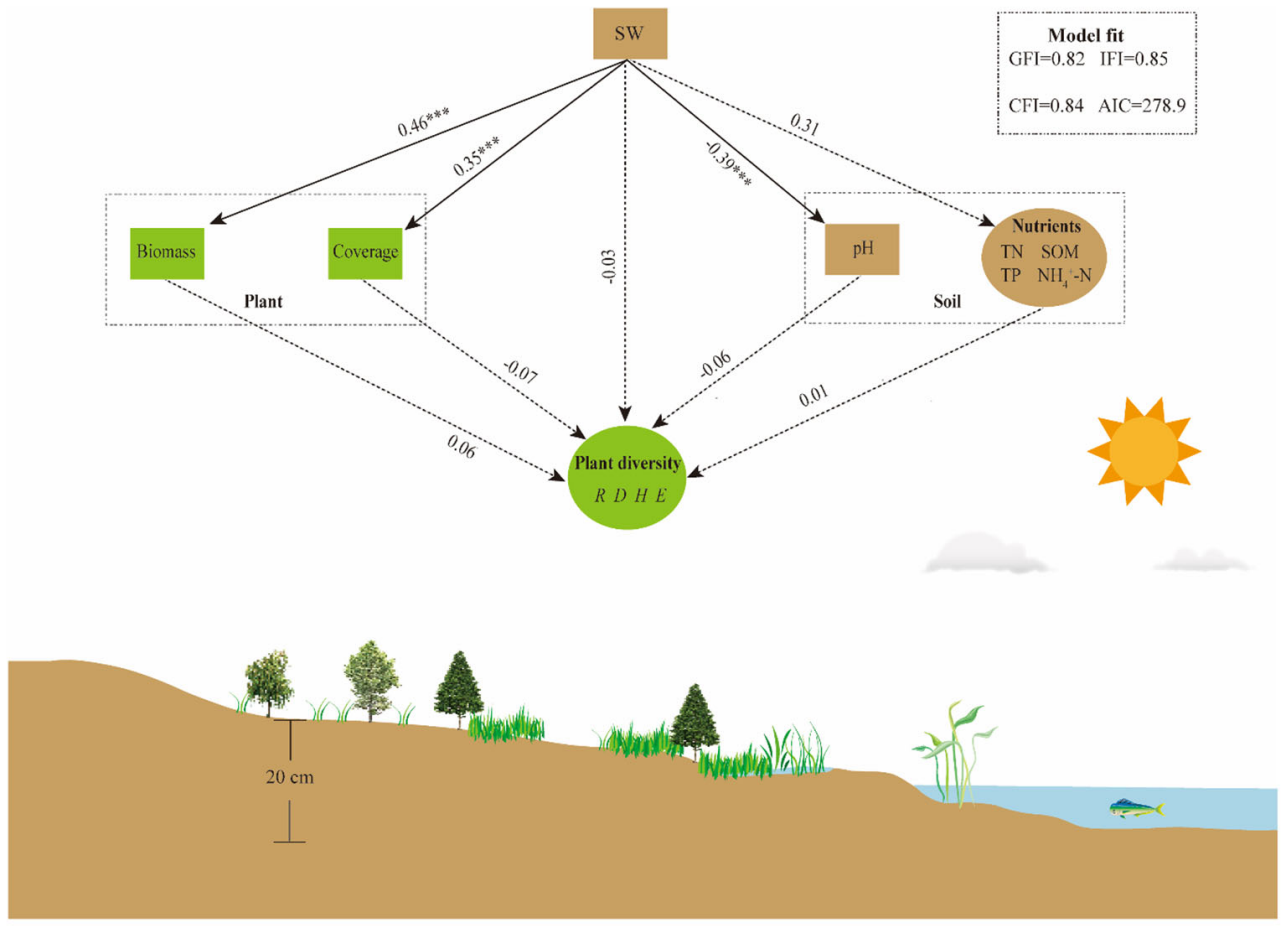
Path analysis results for structural equation modeling. The numbers adjacent to the arrows are standardized path coefficients, with significantly different indicated as ***P < 0.001. Solid and dashed arrows indicate significant and non-significant paths, respectively. SW, soil water content; TN, total nitrogen; SOM, soil organic matter; TP, total phosphorus; NH_4_^+^-N, ammonia nitrogen. *R*: species richness; *D*: Simpson index; *H*: Shannon-Wiener index; *E*: Pielou Evenness index.

To further reveal the effects of environmental factors such as SW and soil nutrients on plant communities, we employed path analysis of structural equation modeling. The results indicated that changes in SW significantly impacted plant coverage, biomass, and soil pH (**Figure 7**). Specifically, SW exhibited a strong positive correlation with plant biomass and coverage. This suggests that increased levels of soil moisture promote the accumulation of plant biomass, which directly influences plant coverage. SW exhibited a strong negative correlation with soil pH, which indicates that changes in SW had a direct impact on pH. However, SW, biomass, coverage, soil pH and nutrients had no significant impact on plant diversity. In summary, SW had a direct impact on plant biomass, coverage and soil pH, while soil nutrients and plant diversity may be impacted by other environmental factors.

## 4 Discussion

Water level fluctuations not only shape the vertical structure of lakeshore wetland plants, but are also a key factor in maintaining the structure and composition of plant communities. In the context of two hydrological years, we studied plant species composition, diversity, aboveground biomass and soil nutrient content at three elevation ranges during the peak growth season of plants in the Erhai lakeshore zone. We aimed to explore the relationship between “hydrological changes - soil properties - plant responses” and the potential influence mechanism. The results indicated that changes in soil water content caused by wet-dry alternation had significant impacts on plant coverage, biomass and soil pH, and that plant diversity gradually increased from low to high elevations.

### 4.1 Influence of flooding on plant communities

Plant diversity, aboveground biomass and coverage are crucial indicators for analyzing plant communities during the wet-dry alternation of lakeshore wetlands. Differences in soil moisture and nutrients across hydrological gradients create diverse habitats, providing various niches that facilitate species coexistence. The niche breadth of lakeshore plants is constrained by flooding, and long-term flooding affects their resource utilization capabilities and distribution ranges (Lou et al., 2018). Only species well adapted to flooding environments can survive, and the resulting homogeneity reduces interspecies competition (Campbell and Keddy, 2022; Kercher and Zedler, 2004), leading to lower plant diversity in low-elevation zones. However, increases in elevation are usually accompanied by changes in resource competition, with competitive species employing drought tolerance and efficient nutrient utilization strategies to dominate (Merlin et al., 2015). These adaptive strategies not only enhance species viability but also increase and maintain plant diversity.

The abundance and diversity of wetland plant species decreased with decreasing elevation under the influence of water level fluctuations (Zhang et al., 2022), consistent with our research findings (**Figure 4**). The study by Qi et al. (2021) also revealed that the numbers of plant species and diversity indices in long-term and periodically flooded areas were significantly lower compared to those in dry areas. Moreover, plant diversity indices and species richness were significantly reduced during the drought year compared to the flooding year (**Figure 4**). Periodic flooding increases soil moisture, nutrient availability, and seed dispersal range, facilitating niche expansion for hygrophytes to obtain more resources and promoting the formation of diverse plant communities (Baschuk et al., 2012; Dai et al., 2019). Under drought stress, only a few drought-tolerant species can prevent water loss by decreasing leaf area and biomass, and seed dispersal is limited, leading to a decrease in overall plant diversity (Xiong et al., 2023). Our results proved that the important values of typical hygrophytes such as *Alternanthera philoxeroides*, *Leersia hexandra*, and *Phragmites australis* were reduced under drought stress (**Figure 3**). Water level fluctuations are the primary drivers affecting plant coverage and biomass in wetlands, and hygrophytes tend to respond to drought-induced survival threats by reducing biomass accumulation and coverage (Wang et al., 2022; Yuan et al., 2017).

### 4.2 Influence of flooding on soil physicochemical properties

Flooding duration is one of the main factors influencing the physicochemical properties of lakeshore soils, and variations in these properties across different elevation ranges can be attributed to fluctuations in water levels. The higher soil water content in low-elevation areas is associated with long-term flooding, while the lower frequency of wet-dry alternation in high-elevation areas results in lower soil water content (Li et al., 2018a; Li et al., 2016). Soil water content is higher in the flooding year than in the drought year (**Figure 5A**), and this change directly affects soil pH and nitrogen content. The pH of alkaline soils is negatively correlated with water content, as moist soils accelerate the dilution of acidic ions (hydrogen ions), with increased moisture causing soil pH to tend toward neutrality (Ding et al., 2019). In addition, low moisture slows the decomposition of soil organic matter, and reduced acid production leads to a higher soil pH (Li et al., 2022). Our results of lower soil organic matter in drought year than in flooding year confirmed this (**Figure 5E**). Previous studies had indicated that wetland soil ammonia nitrogen content increased with flooding time (Hu et al., 2019), and increasing soil pH accelerated ammonia nitrogen volatilization and reduced ammonia nitrogen content (Bai et al., 2005; Rao et al., 1984). The combined effect of reduced soil water content, plant absorption and nitrification decreases ammonia nitrogen availability (Swanson et al., 2017), while nitrate nitrogen is produced through the nitrification process of ammonia nitrogen. Therefore, ammonia nitrogen content influences the potential production of nitrate nitrogen. In the flooding year, rapid decomposition of organic matter and increased inputs of nitrogen sources resulted in significantly higher ammonia and nitrate nitrogen contents in wetland soils than in the drought year (Shen et al., 2020), which is consistent with our findings (**Figures 5C, D**). Therefore, changes in flooding days, soil water content and pH created a new physicochemical balance of the wetland soil environment, with soil water content being the most critical, this conclusion was confirmed by the RDA analysis and SEM results (**Figures 6**, **7**).

### 4.3 Plant-soil interaction and critical factor under different hydrological conditions

Wetland soil exerts a direct and significant impact on plant growth by providing essential nutrients. At low elevations, higher soil water content accelerates the solubilization of soil nutrients and promotes plant root expansion for efficient nutrient uptake, thereby increasing plant biomass and coverage (Zhang et al., 2022). Conversely, drought stress inhibits plant root uptake capability and soil microbial activity, and reduces leaf area, collectively resulting in decreased plant biomass and coverage (Bogati and Walczak, 2022; Eziz et al., 2017). The RDA and SEM results indicated that soil water content was significantly positively correlated with both plant biomass and coverage, suggesting that soil moisture directly affects plant growth (**Figures 6**, **7**). Plant litter is a crucial source of nutrients in wetland soils, and biomass influences nutrient content levels. On one hand, the decomposition of high-biomass plants by soil microbial significantly increases soil nutrient content (Fennessy et al., 2008). On the other hand, under the influence of hydrological processes, faster water flow speeds tend to scour away soil nutrients in low-elevation areas, while in higher elevation areas, nutrients can be retained after undergoing sufficient physicochemical reactions (Zhang et al., 2022). Our results suggested that tall herbaceous plants such as *Ageratina adenophora*, *Symphyotrichum subulatum*, and *Erigeron canadensis* were the dominant species within the range of 1965.6-1966.4m, and their higher biomass returned more nutrients to the soil. Therefore, changes in community composition and diversity within this elevation range are primarily constrained by soil nutrients, with a lower explanatory soil water content (**Figure 6C**).

Based on these results, we infer that interannual variations in hydrological processes lead to changes in plant and soil physicochemical properties at the Erhai lakeshore zone, with soil water content identified as a critical factor affecting plant biomass, coverage, and soil pH. Meanwhile, plant diversity along the lakeshore exhibited a gradual increase from the waterside to upland areas, attributed to niche differentiation, edge effects, and soil physicochemical properties (Emery and Ackerly, 2014). These research results confirmed the close ecological connections and interactions between “hydrological changes - soil properties - plant responses” in wetland ecosystems.

### 4.4 Management implications

The water level of Lake Erhai is low in summer and high in autumn, with distinct wet and dry seasons but uneven precipitation distribution. The rainy season from May to October accounts for more than 85% of the annual precipitation, while the dry season from November to April has relatively little precipitation. Since 2004, the water level of Lake Erhai has been influenced primarily by precipitation and artificial regulation, and climate change has gradually increased the time lag between precipitation and water level (Wen et al., 2021). This change is expected to significantly impact the structure and diversity of plant communities in the lakeshore zone, posing a challenge for the ecological protection and restoration of Lake Erhai.

This study demonstrated that low water levels during extreme drought year significantly decreased plant diversity, biomass, and coverage in the lakeshore zone. It also revealed that soil water content is a critical factor affecting plant communities, with significant differences observed across elevation ranges. Based on the results of this study and management practice needs, we propose the following suggestions: 1) Strengthen water level monitoring and management, and scientifically formulate water level regulation programs to avoid the impacts of extreme droughts and floods; 2) Conduct vegetation restoration projects in the lakeshore zone, and target plant species for planting in different elevation zones; and 3) Implement diversified vegetation restoration and maintenance, and enhance the adaptability of plant communities to water level changes by increasing plant species and optimizing community structure.

## 5 Conclusion

Under the influence of global warming, regional precipitation patterns, distribution patterns of wetland ecosystem species, and productivity will undergo a series of changes. To better understand the chain reactions of climate change on wetland water levels, plants, and soil, we studied the characteristics of plant diversity, coverage, biomass, and soil physicochemical properties across three elevation ranges of the Erhai lakeshore zone during two hydrological years. We explored the interrelationships between “hydrological changes - soil properties - plant responses” under different flooding scenarios. The study showed that changes in soil water content significantly affected plants and soils in the lakeshore zone, with differences in soil-plant relationships across three elevation ranges. Plant communities at lower elevations were directly influenced by soil water content, while those at the highest elevation were influenced by soil nutrients. Plant diversity increases gradually from low to high elevations, and both plant diversity and soil physicochemical properties are generally lower in drought year compared to flooding year. These results profoundly revealed the significant effects of interannual hydrological differences on soils and plants in the lakeshore zone, and provided new insights into the interrelationships between plants and soils across different hydrological gradients. Our findings provide important guidance for developing effective wetland water level management in response to extreme drought events and offer a scientific basis for increasing plant diversity in the lakeshore zone.

## Data Availability

The original contributions presented in the study are included in the article/**Supplementary Material**. Further inquiries can be directed to the corresponding author/s.

## References

[B1] Bai J. H. Hua O. Y. Wei D. Zhu Y. M. Zhang X. L. Wang Q. G. (2005). Spatial distribution characteristics of organic matter and total nitrogen of marsh soils in river marginal wetlands. Geoderma 124, 181–192. doi: 10.1016/j.geoderma.2004.04.012

[B2] Baschuk M. S. Ervin M. D. Clark W. R. Armstrong L. M. Wrubleski D. A. Goldsborough G. L. (2012). Using satellite imagery to assess macrophyte response to water-level manipulations in the Saskatchewan River Delta, Manitoba. Wetlands 32, 1091–1102. doi: 10.1007/s13157-012-0339-z

[B3] Bernal S. von Schiller D. Sabater F. Martí E. (2013). Hydrological extremes modulate nutrient dynamics in mediterranean climate streams across different spatial scales. Hydrobiologia 719, 31–42. doi: 10.1007/s10750-012-1246-2

[B4] Bogati K. Walczak M. (2022). The impact of drought stress on soil microbial community, enzyme activities and plants. Agronomy-Basel 12. doi: 10.3390/agronomy12010189

[B5] Campbell D. Keddy P. (2022). The roles of competition and facilitation in producing zonation along an experimental flooding gradient: A tale of two tails with ten freshwater marsh plants. Wetlands 42. doi: 10.1007/s13157-021-01524-4

[B6] Cao J. Wu Y. Li Z. K. Hou Z. Y. Wu T. H. Chu Z. S.. (2024). Dependence of evolution of cyanobacteria superiority on temperature and nutrient use efficiency in a meso-eutrophic plateau lake. Sci. Total Environ. 927. doi: 10.1016/j.scitotenv.2024.172338 38608897

[B7] Chapin D. M. Paige D. K. (2013). Response of delta vegetation to water level changes in a regulated mountain lake, Washington State, USA. Wetlands 33, 431–444. doi: 10.1007/s13157-013-0401-5

[B8] Chen J. Wu C. (2020). Evaluation of ecological sensitivity in Erhai Lake basin, southwest China. IOP. Conf. Series: Earth Environ. Sci. 612, 12072. doi: 10.1088/1755-1315/612/1/012072

[B9] Crisman T. L. Alexandridis T. K. Zalidis G. C. Takavakoglou V. (2014). Phragmites distribution relative to progressive water level decline in Lake Koronia, Greece. Ecohydrology 7, 1403–1411. doi: 10.1002/eco.1466

[B10] Dai X. Wan R. R. Yang G. S. Wang X. L. Xu L. G. Li Y. Y.. (2019). Impact of seasonal water-level fluctuations on autumn vegetation in Poyang Lake wetland, China. Front. Earth. Sci. 13, 398–409. doi: 10.1007/s11707-018-0731-y

[B11] Dali Bai Autonomous Prefecture People’s Government (DBAPPG). (2023). Proposal for deliberation on the minimum operating water level of Erhai Lake in the special year. Available online at: https://www.dali.gov.cn/dlrmzf/c101530/202306/03cfc4fc80c6435ab5e7343e6c9e3cf0.shtml (Accessed 2 June 2023).

[B12] Delgado-Baquerizo M. Maestre F. T. Gallardol A. Bowker M. A. Wallenstein M. D. Quero J. L.. (2013). Decoupling of soil nutrient cycles as a function of aridity in global drylands. Nature 502, 672–67+. doi: 10.1038/nature12670 24172979

[B13] Deng F. Wang X. L. Cai X. B. Li E. H. Jiang L. Z. Li H.. (2014). Analysis of the relationship between inundation frequency and wetland vegetation in Dongting Lake using remote sensing data. Ecohydrology 7, 717–726. doi: 10.1002/eco.1393

[B14] Ding C. F. Du S. Y. Ma Y. B. Li X. G. Zhang T. L. Wang X. X. (2019). Changes in the pH of paddy soils after flooding and drainage: Modeling and validation. Geoderma 337, 511–513. doi: 10.1016/j.geoderma.2018.10.012

[B15] Emery N. C. Ackerly D. D. (2014). Ecological release exposes genetically based niche variation. Ecol. Lett. 17, 1149–1157. doi: 10.1111/ele.12321 25040103

[B16] Eziz A. Yan Z. B. Tian D. Han W. X. Tang Z. Y. Fang J. Y. (2017). Drought effect on plant biomass allocation: A meta-analysis. Ecol. Evolution. 7, 11002–11010. doi: 10.1002/ece3.3630 PMC5743700 29299276

[B17] Fan H. X. Xu L. G. Wang X. L. Jiang J. H. Feng W. J. You H. L. (2019). Relationship between vegetation community distribution patterns and environmental factors in typical wetlands of Poyang Lake, China. Wetlands 39, S75–S87. doi: 10.1007/s13157-017-0903-7

[B18] Fan Y. Miguez-Macho G. Jobbágy E. G. Jackson R. B. Otero-Casal C. (2017). Hydrologic regulation of plant rooting depth. Proc. Natl. Acad. Sci. U. S. A. 114, 10572–10577. doi: 10.1073/pnas.1712381114 28923923 PMC5635924

[B19] Feng W. J. Santonja M. Bragazza L. Buttler A. (2020). Shift in plant-soil interactions along a lakeshore hydrological gradient. Sci. Total Environ. 742. doi: 10.1016/j.scitotenv.2020.140254 32721708

[B20] Fennessy M. S. Rokosch A. Mack J. J. (2008). Patterns of plant decomposition and nutrient cycling in natural and created wetlands. Wetlands 28, 300–310. doi: 10.1672/06-97.1

[B21] Fluet-Chouinard E. Stocker B. D. Zhang Z. Malhotra A. Melton J. R. Poulter B.. (2023). Extensive global wetland loss over the past three centuries. Nature 614, 281–28+. doi: 10.1038/s41586-022-05572-6 36755174

[B22] Fu H. Wang X. Y. Ge D. B. Li W. Tan X. Y. Yuan G. X.. (2022). Human activities uncouple the cascading effects of hydrological gradients on plant diversity and ecosystem functions in the Lake Dongting wetland. Ecohydrology 15. doi: 10.1002/eco.2359

[B23] Fu H. Yuan G. Cao T. Zhong J. Zhang X. Guo L.. (2013). Succession of submerged macrophyte communities in relation to environmental change in Lake Erhai over the past 50 years. J. Lake Sci. 25, 854–861. doi: 10.18307/2013.0609

[B24] Gong F. Luo L. Li H. Chen L. Zhang R. Wu G.. (2023). Quantitative assessment of water quality improvement by reducing external loadings at Lake Erhai, Southwest China. Int. J. Environ. Res. Public. Health 20. doi: 10.3390/ijerph20065038 PMC10048958 36981948

[B25] Hertling U. M. Lubke R. A. (1999). Indigenous and Ammophila arenaria-dominated dune vegetation on the South African Cape coast. Appl. Veg. Sci. 2, 157–168. doi: 10.2307/1478979

[B26] Hu W. F. Zhang W. L. Zhang L. H. Lin X. B. Tong C. Lai D. Y. F.. (2019). Short-term changes in simulated inundation frequency differentially affect inorganic nitrogen, nitrification, and denitrification in estuarine marshes. Ecol. Indic. 107. doi: 10.1016/j.ecolind.2019.105571

[B27] Ji T. (2005). Comparison on determining the organic matter contents in the soils by different heating methods in the potassium dichromate-volumetric method. Acta Agriculturae Zhejiangensis 17, 311–313.

[B28] Kercher S. M. Zedler J. B. (2004). Flood tolerance in wetland angiosperms: A comparison of invasive and noninvasive species. Aquat Bot. 80, 89–102. doi: 10.1016/j.aquabot.2004.08.003

[B29] Lan Z. C. Chen Y. S. Li L. Li F. Jin B. S. Chen J. K. (2019). Testing mechanisms underlying elevational patterns of lakeshore plant community assembly in Poyang Lake, China. J. Plant Ecol. 12, 438–447. doi: 10.1093/jpe/rty027

[B30] Lawniczak A. E. Zbierska J. Choinski A. Szczepaniak W. (2010). Response of emergent macrophytes to hydrological changes in a shallow lake, with special reference to nutrient cycling. Hydrobiologia 656, 243–254. doi: 10.1007/s10750-010-0436-z

[B31] Li F. Hu J. Y. Xie Y. H. Yang G. S. Hu C. Chen X. S.. (2018a). Foliar stoichiometry of carbon, nitrogen, and phosphorus in wetland sedge *Carex brevicuspis* along a small-scale elevation gradient. Ecol. Indic. 92, 322–329. doi: 10.1016/j.ecolind.2017.04.059

[B32] Li W. Ma L. Zang Z. Gao J. Li J. (2018b). Construction of ecological security patterns based on ecological red line in Erhai Lake Basin of southwestern China. J. Beijing Forestry Univ. 40, 85–95.

[B33] Li X. Song B. Li F. Zeng J. Hou Z. Xie Y.. (2016). Population distribution patterns and growing status of Triarrhena lutarioriparia along a gentle elevation gradient of Lake Dongting wetlands. J. Lake Sci. 28, 1039–1046.

[B34] Li Y. Wang X. He C. G. Jiang H. B. Sheng L. X. (2022). Multi-environment factors dominate plant community structure and diversity in an ombrotrophic bog: The water level is the main regulating mechanism. Front. Environ. Sci. 10. doi: 10.3389/fenvs.2022.1032068

[B35] Lili W. Kebin Z. Jin C. Zhongqiu C. Jian L. I. U. (2011). Siertan wetland vegetation niche of Yanchi County in semiarid areas. Bull. Soil Water Conserv. 31, 68.

[B36] Lin S. S. Shen S. L. Zhou A. N. Lyu H. M. (2020). Sustainable development and environmental restoration in Lake Erhai, China. J. Clean. Prod. 258. doi: 10.1016/j.jclepro.2020.120758

[B37] Liu S. L. Hou X. Y. Yang M. Cheng F. Y. Coxixo A. Wu X.. (2018). Factors driving the relationships between vegetation and soil properties in the Yellow River Delta, China. Catena 165, 279–285. doi: 10.1016/j.catena.2018.02.004

[B38] Liu Y. Wang L. Liu H. Wang W. Liang C. Yang J.. (2015). Comparison of carbon sequestration ability and effect of elevation in fenced wetland plant communities of the Xilin River Floodplains: A model case study. River Res. Appl. 31, 858–866. doi: 10.1002/rra.2777

[B39] Lou Y. J. Gao C. Y. Pan Y. W. Xue Z. S. Liu Y. Tang Z. H.. (2018). Niche modelling of marsh plants based on occurrence and abundance data. Sci. Total Environ. 616, 198–207. doi: 10.1016/j.scitotenv.2017.10.300 29121575

[B40] Luo F. L. Jiang X. X. Li H. L. Yu F. H. (2016). Does hydrological fluctuation alter impacts of species richness on biomass in wetland plant communities? J. Plant Ecol. 9, 434–441. doi: 10.1093/jpe/rtv065

[B41] Ma M. Y. Zhu Y. J. Wei Y. Y. Zhao N. N. (2021). Soil nutrient and vegetation diversity patterns of alpine wetlands on the Qinghai-Tibetan Plateau. Sustainability 13. doi: 10.3390/su13116221

[B42] Merlin A. Bonis A. Damgaard C. F. Mesléard F. (2015). Competition is a strong driving factor in wetlands, peaking during drying out periods. PloS One 10. doi: 10.1371/journal.pone.0130152 PMC4468187 26075597

[B43] Nishihiro J. Araki S. Fujiwara N. Washitani I. (2004). Germination characteristics of lakeshore plants under an artificially stabilized water regime. Aquat. Bot. 79, 333–343. doi: 10.1016/j.aquabot.2004.05.005

[B44] Peng J. Y. Hou Z. Y. Yuan J. Wu Y. Yang K. L. Lei B. K.. (2024). The storm runoff management strategy based on agricultural ditch nutrient loss characteristics in Erhai lake, China. J. Contam Hydrol. 261. doi: 10.1016/j.jconhyd.2024.104305 38301313

[B45] Qi Q. Zhang D. J. Zhang M. Y. Tong S. Z. An Y. Wang X. H.. (2021). Hydrological and microtopographic effects on community ecological characteristics of *Carex schmidtii* tussock wetland. Sci. Total Environ. 780. doi: 10.1016/j.scitotenv.2021.146630 34030303

[B46] Qin J. L. Sun Y. Y. Qiu X. T. Liu H. R. Zhang E. Z. Mao X. Q. (2021). Distribution pattern simulation of multiple emergent plants in river riparian zones. River Res. Appl. 37, 1180–1190. doi: 10.1002/rra.3721

[B47] Rao P. S. C. Jessup R. E. Reddy K. R. (1984). Simulation of nitrogen dynamics in flooded soils. Soil Sci. 138, 54–62. doi: 10.1097/00010694-198407000-00009

[B48] Ren Q. Yuan J. H. Wang J. P. Liu X. Ma S. L. Zhou L. Y.. (2022). Water level has higher influence on soil organic carbon and microbial community in Poyang Lake wetland than vegetation type. Microorganisms 10. doi: 10.3390/microorganisms10010131 PMC8779464 35056580

[B49] Riis T. Hawes I. (2002). Relationships between water level fluctuations and vegetation diversity in shallow water of New Zealand lakes. Aquat. Bot. 74, 133–148. doi: 10.1016/s0304-3770(02)00074-8

[B50] Rücker K. Schrautzer J. (2010). Nutrient retention function of a stream wetland complex - A high-frequency monitoring approach. Ecol. Eng. 36, 612–622. doi: 10.1016/j.ecoleng.2008.12.035

[B51] Shen R. C. Lan Z. C. Chen Y. S. Leng F. Jin B. S. Fang C. M.. (2019). The effects of flooding regimes and soil nutrients on lakeshore plant diversity in a pristine lake and a human managed lake in subtropical China. J. Freshw. Ecol. 34, 757–769. doi: 10.1080/02705060.2019.1687340

[B52] Shen R. C. Lan Z. C. Huang X. Y. Chen Y. S. Hu Q. W. Fang C. M.. (2020). Soil and plant characteristics during two hydrologically contrasting years at the lakeshore wetland of Poyang Lake, China. J. Soils Sediments 20, 3368–3379. doi: 10.1007/s11368-020-02638-8

[B53] Sollie S. Verhoeven J. T. A. (2008). Nutrient cycling and retention along a littoral gradient in a Dutch shallow lake in relation to water level regime. Water Air Soil pollut. 193, 107–121. doi: 10.1007/s11270-008-9671-6

[B54] Song T. J. An Y. Tong S. Z. Zhang W. Wang X. Wang L.. (2023). Soil water conditions together with plant nitrogen acquisition strategies control vegetation dynamics in semi-arid wetlands undergoing land management changes. Catena 227. doi: 10.1016/j.catena.2023.107115

[B55] Strong W. L. (2016). Biased richness and evenness relationships within Shannon-Wiener index values. Ecol. Indic. 67, 703–713. doi: 10.1016/j.ecolind.2016.03.043

[B56] Sun B. Y. Jiang M. Han G. X. Zhang L. W. Zhou J. Bian C. Y.. (2022). Experimental warming reduces ecosystem resistance and resilience to severe flooding in a wetland. Sci. Adv. 8. doi: 10.1126/sciadv.abl9526 PMC8791607 35080980

[B57] Swanson W. De Jager N. R. Strauss E. Thomsen M. (2017). Effects of flood inundation and invasion by Phalaris arundinacea on nitrogen cycling in an Upper Mississippi River floodplain forest. Ecohydrology 10. doi: 10.1002/eco.1877

[B58] Thiet R. K. (2002). Diversity comparisons between diked and undiked coastal freshwater marshes on Lake Erie during a high-water year. J. Gt. Lakes Res. 28, 285–298. doi: 10.1016/s0380-1330(02)70584-4

[B59] Wan R. R. Dai X. Shankman D. (2019). Vegetation response to hydrological changes in Poyang Lake, China. Wetlands 39, S99–S112. doi: 10.1007/s13157-018-1046-1

[B60] Wang J. Song Y. H. Ge B. C. Zhou Y. (2023b). Dynamic spatiotemporal land use evolution in China’s plateau lake basins in response to landscape ecological sensitivity. Sustainability 15. doi: 10.3390/su152015020

[B61] Wang X. Wang H. L. Wang H. Y. Guo W. X. Zhai H. Y. Zhang X. K. (2022). Responses of lakeshore herbaceous plant guilds to altered water level fluctuations in Yangtze floodplain lakes, China. Ecol. Indic. 145. doi: 10.1016/j.ecolind.2022.109714

[B62] Wang S. R. Zhang L. Ni L. Y. Zhao H. C. Jiao L. X. Yang S. W.. (2015). Ecological degeneration of the Erhai Lake and prevention measures. Environ. Earth Sci. 74, 3839–3847. doi: 10.1007/s12665-015-4433-4

[B63] Wang H. L. Zhang X. K. Xu Y. W. Wang H. Y. Song M. Y. Shen Y. B. (2023a). Ecological regulation of water level should be combined with seed supplementation for lakeshore *Carex* community restoration in Yangtze-disconnected lakes. Sci. Total Environ. 897. doi: 10.1016/j.scitotenv.2023.165358 37419353

[B64] Wei C. Z. Yu Q. Bai E. Lü X. T. Li Q. Xia J. Y.. (2013). Nitrogen deposition weakens plant-microbe interactions in grassland ecosystems. Glob. Change Biol. 19, 3688–3697. doi: 10.1111/gcb.12348 23925948

[B65] Wen Z. H. Ma Y. W. Wang H. Cao Y. Yuan C. B. Ren W. J.. (2021). Water level regulation for eco-social services under climate change in Erhai Lake over the past 68 years in China. Front. Environ. Sci. 9. doi: 10.3389/fenvs.2021.697694

[B66] Xiong Y. Mo S. H. Wu H. P. Qu X. Y. Liu Y. Y. Zhou L. (2023). Influence of human activities and climate change on wetland landscape pattern-A review. Sci. Total Environ. 879. doi: 10.1016/j.scitotenv.2023.163112 36966825

[B67] Yang T. Yuan C. Cao T. Wen Z. Chou Q. Mao Y.. (2021). Preliminary study on recovery and optimization of submerged macrophyte community in Lake Erhai, China. J. Lake Sci. 33, 1777–1787. doi: 10.18307/2021.0614

[B68] Yu J. M. Wang X. T. Yang S. X. Guo Y. Y. Liu M. Y. Xi M. (2023). Divergent response of blue carbon components to wetland types and hydrological effects in typical estuarine wetlands of Jiaozhou Bay, China. J. Environ. Manage. 347. doi: 10.1016/j.jenvman.2023.119233 37812903

[B69] Yuan J. Cao J. Liao W. X. Zhu F. Hou Z. Y. Chu Z. S. (2024). Effects of vegetation cover varying along the hydrological gradient on microbial community and n-cycling gene abundance in a plateau lake littoral zone. Processes 12. doi: 10.3390/pr12061276

[B70] Yuan S. B. Yang Z. D. Liu X. Q. Wang H. Z. (2017). Key parameters of water level fluctuations determining the distribution of *Carex* in shallow lakes. Wetlands 37, 1005–1014. doi: 10.1007/s13157-017-0934-0

[B71] Zhang X. Hu Z. Chu S. (2005). Methods for measuring soil water content: A review. J. Soil Sci. 36, 118–123.

[B72] Zhang X. K. Liu X. Q. Wang H. Z. (2015). Effects of water level fluctuations on lakeshore vegetation of three subtropical floodplain lakes, China. Hydrobiologia 747, 43–52. doi: 10.1007/s10750-014-2121-0

[B73] Zhang X. K. Qin H. M. Wang H. L. Wan A. Liu G. H. (2018). Effects of water level fluctuations on root architectural and morphological traits of plants in lakeshore areas of three subtropical floodplain lakes in China. Environ. Sci. pollut. Res. 25, 34583–34594. doi: 10.1007/s11356-018-3429-5 30315531

[B74] Zhang Q. J. Wang Z. S. Xia S. X. Zhang G. S. Li S. X. Yu D. K.. (2022). Hydrologic-induced concentrated soil nutrients and improved plant growth increased carbon storage in a floodplain wetland over wet-dry alternating zones. Sci. Total Environ. 822. doi: 10.1016/j.scitotenv.2022.153512 35101500

[B75] Zhao J. X. Li R. C. Li X. Tian L. H. (2017). Environmental controls on soil respiration in alpine meadow along a large altitudinal gradient on the central Tibetan Plateau. Catena 159, 84–92. doi: 10.1016/j.catena.2017.08.007

