# Supplementary material for: Interannual hydrological changes affect plant communities across different elevation zones in plateau lakeshores: insights from Lake Erhai
Source: Front Plant Sci. 2024 Nov 14;15:1439772. doi: 10.3389/fpls.2024.1439772 (PMC11602318; doi:10.3389/fpls.2024.1439772)
Supplement: Supplementary file 1 [file Table1.docx]

Appendix A.1: Distributions and importance value index changes of dominant species at different elevations. Values are species important value means and standard errors (mean±SE).

| Dominant species | Families | Genera | Year | Important values of species at different elevations | | |
| --- | --- | --- | --- | --- | --- | --- |
|  |  |  |  | 1965.0-1965.3 | 1965.3-1965.6 | 1965.6-1966.4 |
| *Polypogon fugax* | Poaceae | Polypogon | 2022 | 0.02±0.04 | 0.04±0.06 | 0.01±0.02 |
|  |  |  | 2023 | 0.02±0.04 | 0.05±0.13 | 0.02±0.16 |
| *Paspalum distichum* | Poaceae | Paspalum | 2022 | 0.05±0.10 | 0.01±0.02 | 0.01±0.02 |
|  |  |  | 2023 | 0.04±0.12 | 0.02±0.07 | 0.01±0.04 |
| *Hemarthria sibirica* | Poaceae | Hemarthria | 2022 | 0.04±0.07 | 0.04±0.06 | 0.03±0.07 |
|  |  |  | 2023 | 0.02±0.04 | 0.03±0.10 | 0.05±0.11 |
| *Leersia hexandra* | Poaceae | Leersia | 2022 | 0.11±0.12 | 0.01±0.04 | 0.01±0.05 |
|  |  |  | 2023 | 0.05±0.17 | 0.03±0.06 | 0.02±0.04 |
| *Phragmites australis* | Poaceae | Phragmites | 2022 | 0.01±0.03 | 0.12±0.20 | 0.11±0.19 |
|  |  |  | 2023 | 0.02±0.05 | 0.12±0.20 | 0.24±0.30 |
| *Capillipedium parviflorum* | Poaceae | Capillipedium | 2022 | 0.7±0.10 | 0.03±0.06 | 0.01±0.03 |
|  |  |  | 2023 | — | 0.02±0.08 | 0.02±0.05 |
| *Erigeron canadensis* | Compositae | Erigeron | 2022 | 0.02±0.04 | 0.06±0.06 | 0.06±0.05 |
|  |  |  | 2023 | 0.01±0.01 | 0.04±0.05 | 0.09±0.16 |
| *Bidens pilosa* | Compositae | Bidens | 2022 | 0.01±0.03 | 0.03±0.04 | 0.03±0.04 |
|  |  |  | 2023 | 0.03±0.10 | 0.02±0.05 | 0.02±0.06 |
| *Bidens tripartita* | Compositae | Bidens | 2022 | 0.07±0.09 | 0.08±0.08 | 0.06±0.10 |
|  |  |  | 2023 | 0.01±0.03 | 0.00±0.01 | 0.01±0.02 |
| *Ageratina adenophora* | Compositae | Ageratina | 2022 | 0.002±0.01 | 0.06±0.10 | 0.05±0.05 |
|  |  |  | 2023 | 0.01±0.04 | 0.10±0.17 | 0.11±0.18 |
| *Symphyotrichum subulatum* | Compositae | Symphyotrichum | 2022 | 0.01±0.02 | 0.01±0.02 | 0.03±0.05 |
|  |  |  | 2023 | 0.01±0.01 | 0.01±0.02 | 0.01±0.02 |
| *Alternanthera philoxeroides* | Amaranthaceae | Alternanthera | 2022 | 0.47±0.17 | 0.22±0.20 | 0.14±0.24 |
|  |  |  | 2023 | 0.39±0.22 | 0.22±0.22 | 0.12±0.18 |
| *Polygonum hydropiper* | Polygonaceae | Persicaria | 2022 | 0.04±0.05 | 0.02±0.04 | 0.02±0.07 |
|  |  |  | 2023 | 0.03±0.05 | 0.02±0.06 | 0.02±0.07 |
| *Urtica atrichocaulis* | Urticaceae | Urtica | 2022 | 0.01±0.02 | 0.03±0.04 | 0.03±0.05 |
|  |  |  | 2023 | — | 0.001±0.01 | — |
| *Equisetum ramosissimum* | Equisetaceae | Equisetum | 2022 | 0.02±0.03 | 0.03±0.06 | 0.02±0.03 |
|  |  |  | 2023 | 0.001±0.002 | 0.004±0.01 | — |

Appendix A.2: Longitude and latitude coordinates of sampling plots at different elevation ranges in the Erhai lakeshore zone. Corresponding to Figure 1.

| Elevation range | Longitude | Latitude |
| --- | --- | --- |
| 1965.0-1965.3m |  |  |
| 1 | 100.206672 | 25.69049 |
| 2 | 100.203599 | 25.7038 |
| 3 | 100.200146 | 25.70875 |
| 4 | 100.171549 | 25.73793 |
| 5 | 100.147583 | 25.83005 |
| 6 | 100.154849 | 25.85267 |
| 7 | 100.095689 | 25.93665 |
| 8 | 100.114398 | 25.93833 |
| 9 | 100.119747 | 25.93779 |
| 10 | 100.136282 | 25.93616 |
| 11 | 100.13758 | 25.93844 |
| 12 | 100.139809 | 25.94084 |
| 13 | 100.146891 | 25.96352 |
| 14 | 100.217797 | 25.83209 |
| 15 | 100.220582 | 25.85339 |
| 16 | 100.271913 | 25.68415 |
| 17 | 100.265414 | 25.70075 |
| 18 | 100.28426 | 25.66362 |
| 19 | 100.278645 | 25.64165 |
| 20 | 100.229743 | 25.61539 |
| 1965.3-1965.6m |  |  |
| 1 | 100.209615 | 25.67536 |
| 2 | 100.206971 | 25.68792 |
| 3 | 100.16212 | 25.74293 |
| 4 | 100.153851 | 25.7518 |
| 5 | 100.152715 | 25.75286 |
| 6 | 100.148225 | 25.76561 |
| 7 | 100.151548 | 25.83848 |
| 8 | 100.15524 | 25.87098 |
| 9 | 100.132744 | 25.87532 |
| 10 | 100.124249 | 25.89663 |
| 11 | 100.121649 | 25.90387 |
| 12 | 100.148362 | 25.96488 |
| 13 | 100.152492 | 25.9645 |
| 14 | 100.2167 | 25.89249 |
| 15 | 100.254952 | 25.71069 |
| 16 | 100.289597 | 25.65979 |
| 17 | 100.268065 | 25.62365 |
| 18 | 100.268013 | 25.62271 |
| 19 | 100.212011 | 25.66163 |
| 20 | 100.213627 | 25.64973 |
| 1965.6-1966.4m |  |  |
| 1 | 100.209483 | 25.67598 |
| 2 | 100.209965 | 25.67286 |
| 3 | 100.207174 | 25.68584 |
| 4 | 100.20521 | 25.70135 |
| 5 | 100.162854 | 25.74247 |
| 6 | 100.160989 | 25.74295 |
| 7 | 100.153577 | 25.75091 |
| 8 | 100.147326 | 25.83286 |
| 9 | 100.157935 | 25.85891 |
| 10 | 100.105344 | 25.92454 |
| 11 | 100.105582 | 25.93068 |
| 12 | 100.133495 | 25.9257 |
| 13 | 100.215513 | 25.84149 |
| 14 | 100.217599 | 25.84608 |
| 15 | 100.263437 | 25.61871 |
| 16 | 100.21579 | 25.64208 |
| 17 | 100.224159 | 25.62655 |
| 18 | 100.21328 | 25.64913 |

Appendix A.3: Results and methods of statistical analysis of soil physicochemical properties at different elevation ranges in the lakeshore zone of Lake Erhai. SW: soil water content; NH_4_^+^-N: ammonia nitrogen; NO_3_^−^-N: nitrate nitrogen; SOM: soil organic matter; TP: total phosphorus; TN: total nitrogen. The t-test indicates that the data are normally distributed, while the u-test is non-normally distributed. Corresponding to Figure 5.

|  | Mean | Standard deviation | Coefficient of variation | Statistical test |
| --- | --- | --- | --- | --- |
| 1965.0-1965.3m |  |  |  |  |
| SW (%) | 36.25591 | 9.381827406 | 0.258766813 | t-test |
| pH | 7.74875 | 0.458672485 | 0.059193094 | u-test |
| NH_4_^+^-N (mg/kg) | 97.84991 | 31.83351298 | 0.325330033 | t-test |
| NO_3_^−^-N (mg/kg) | 17.18305 | 12.32038496 | 0.71700817 | u-test |
| SOM (g/kg) | 52.5407 | 24.00488036 | 0.456881635 | u-test |
| TP (mg/kg) | 872.2105 | 255.5158764 | 0.292952067 | u-test |
| TN (%) | 0.331613 | 0.144151099 | 0.434697118 | u-test |
| C/N ratio | 14.58928 | 5.914661952 | 0.405411572 | u-test |
| 1965.3-1965.6m |  |  |  |  |
| SW (%) | 30.68782 | 8.541858243 | 0.278346835 | t-test |
| pH | 7.58675 | 0.58843725 | 0.077561176 | u-test |
| NH_4_^+^-N (mg/kg) | 93.82386 | 35.97299911 | 0.383409925 | u-test |
| NO_3_^−^-N (mg/kg) | 18.90409 | 13.241537 | 0.700458759 | u-test |
| SOM (g/kg) | 54.68036 | 24.70493846 | 0.451806424 | u-test |
| TP (mg/kg) | 882.7796 | 238.7344172 | 0.270434912 | t-test |
| TN (%) | 0.339702 | 0.128090881 | 0.377068543 | u-test |
| C/N ratio | 11.7259 | 1.942958609 | 0.165697975 | u-test |
| 1965.6-1966.4m |  |  |  |  |
| SW (%) | 27.5386 | 7.463306003 | 0.271012564 | t-test |
| pH | 7.928056 | 0.501846985 | 0.063300135 | t-test |
| NH_4_^+^-N (mg/kg) | 95.9604 | 31.32145413 | 0.326399772 | t-test |
| NO_3_^−^-N (mg/kg) | 15.64675 | 14.64169709 | 0.935765868 | u-test |
| SOM (g/kg) | 42.46706 | 30.36974378 | 0.715136443 | u-test |
| TP (mg/kg) | 1054.874 | 403.4049047 | 0.382420062 | u-test |
| TN (%) | 0.267258 | 0.187342026 | 0.700977369 | u-test |
| C/N ratio | 13.66578 | 2.767303887 | 0.20249879 | t-test |
